# Osimertinib showed efficacy on contralateral multiple ground-glass nodules after segmentectomy for lung adenocarcinoma harboring primary EGFR-T790M mutation: a case report and review of the literature

**DOI:** 10.1186/s13019-022-02071-7

**Published:** 2022-12-19

**Authors:** Haijun Dong, Jianbin Zhang, Weiwei Min, Qibin Shen

**Affiliations:** 1 Department of Thoracic Surgery, Huzhou Central Hospital, Affiliated Central Hospital of HuZhou University, 1558 Third Ring North Road, Huzhou, 313000 Zhejiang China; 2 Department of Pneumology, Huzhou Central Hospital, Affiliated Central Hospital of HuZhou University, 1558 Third Ring North Road, Huzhou, 313000 Zhejiang China

**Keywords:** Multiple ground-glass nodules (mGGNs), Synchronous multiple primary lung cancer (SMPLC), Epidermal growth factor receptor-tyrosine kinase inhibitor (EGFR-TKI)

## Abstract

**Background:**

Multiple ground-glass nodules (mGGNs) in the lung has been defined as synchronous multiple primary lung cancer (SMPLC), it is has been very difficult challenging to differentiate SMPLC from intrapulmonary metastases, and its treatment remains controversial.

**Case presentation:**

We report a case simultaneously involving mGGNs and lung adenocarcinoma harboring primary EGFR-T790M mutation, in which the patient underwent the radical resection of lesions in the left upper lung, and continued the osimertinib treatment for the residual mGGNs in all lobes of the right lung. These mGGNs displayed different responses to osimertinib.

**Conclusions:**

We reported a successful strategy on the postoperative treatment for mGGNs. For those that cannot be completely resected, the chemotherapy, radiotherapy, stereotactic body radiation therapy, immunotherapy and targeted therapy have been performed instead. The EGFR-TKI therapy strategy showed significant advantages, but how to achieve even better therapeutic effect needs more researches.

## Introduction

Ground-glass nodule has often been detected in the chest CT, it might be caused by various illnesses like inflammation, fibrosis, interstitial diseases, or neoplastic transformation of lung.

Multiple ground-glass nodules (mGGNs) occur when over two nodules are found in a single patient at the same time [1]. The mGGNs in the lung have been defined as synchronous multiple primary lung cancer (SMPLC) [2, 3], it is challenging to differentiate SMPLC from intrapulmonary metastases, and the treatment strategy remains controversial [4]. Surgical treatment is usually the first choice, but numerous GGNs patients are not eligible for having a complete surgical resection. In previous studies on clinical innovations, the chemotherapy [5, 6], radiotherapy [7, 8], stereotactic body radiation therapy (SBRT) [9, 10], immunotherapy [11] and targeted therapy [12–15] have been reported to perform, but no consensus results have been obtained yet. As a result, it is meaningful to explore more effective treatments for GGNs.

## Case presentation

**Fig. 1:**
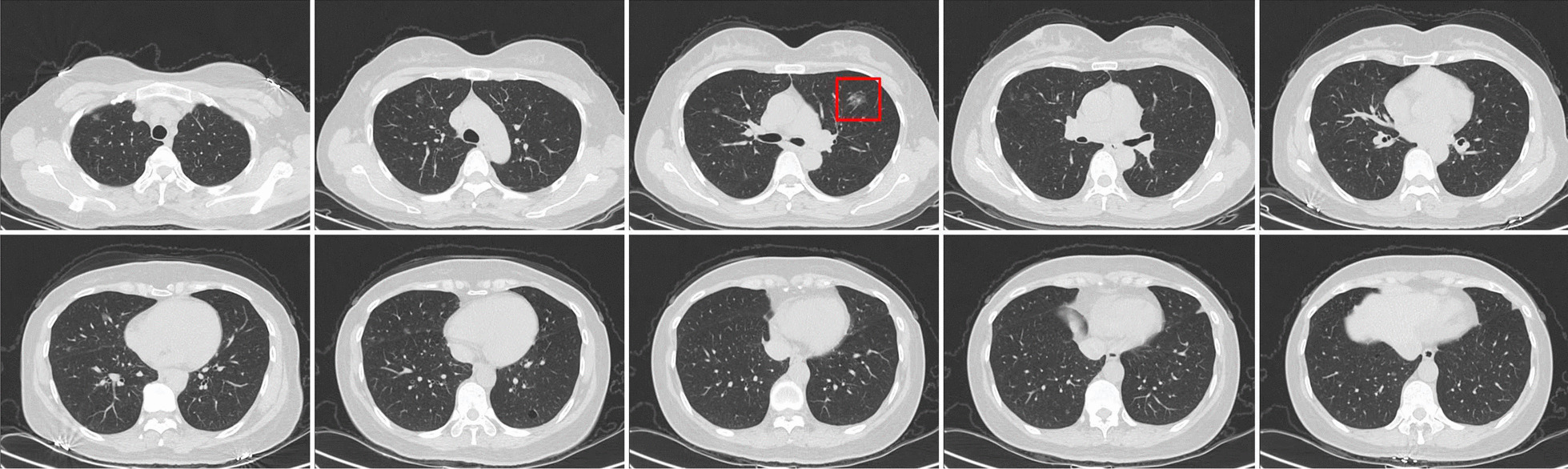
The preoperative computed tomography scanning. A 1.5-cm diameter irregularly shaped lesion in the left upper lung, and mGGNs in all lobes of the right lung

**Fig. 2:**
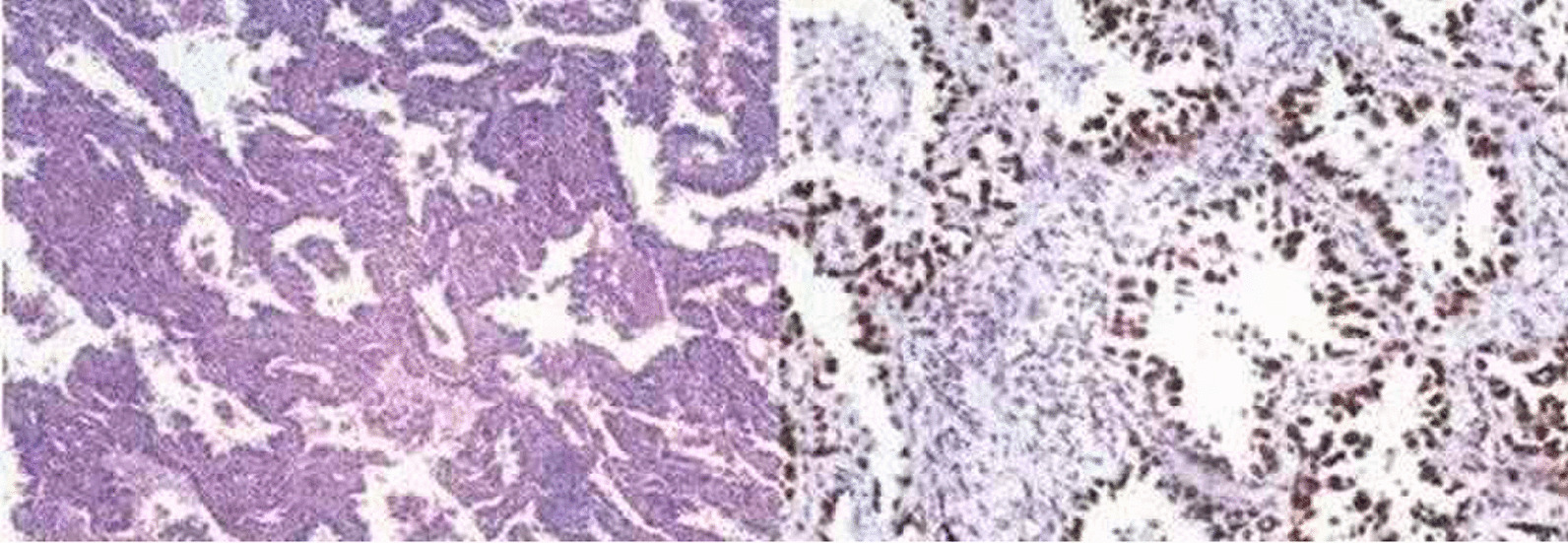
The postoperative pathology. Acinar pulmonary adenocarcinoma with negative resected lymph nodes

**Fig. 3:**
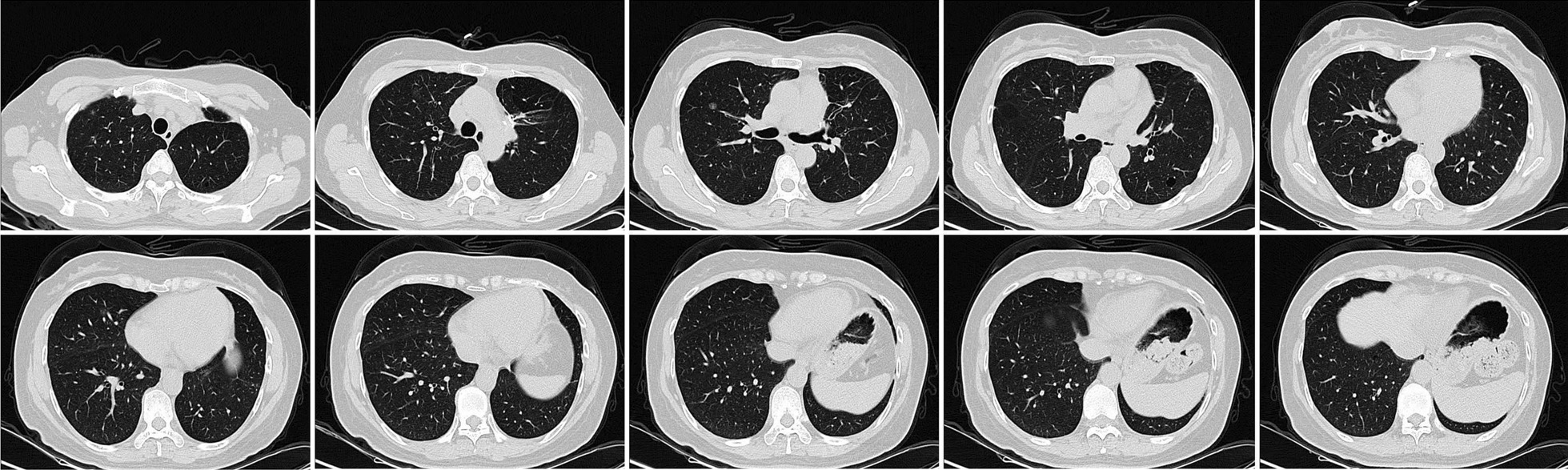
The postoperative computed tomography scanning. After 28 months of target therapy, most mGGNs in the right lung were completely absorbed, only one GGN in the right upper lung was radiologically same as before

In March 2019, a 50-year-old asymptomatic never-smoker woman was admitted to our hospital due to bilateral mGGNs detected on her routine health checkup. She had no significant medical history for herself or family medical history. The chest's computed tomography revealed a 1.5 cm diameter of irregularly shaped lesion in the left upper lung, and 28 ground-glass nodules in all lobes of the right lung (Fig. 1). Systemic examination including the abdominal CT scan, brain MRI, bone scintigraphy, and else, were individually performed for the exclusion of distant metastases Then, we suggested a lung biopsy, but the patient refused. She was then diagnosed with mGGNs, as SMPLC being the most likely, but intrapulmonary metastasis could not be ruled out, either. We selected an isolated nodular lesion in the left lung that could be resected surgically. In March 2019, this patient underwent segmentectomy of left S^(1+2+3)^ and systematic lymph node dissection. Postoperative specimen pathological examination verified the disease as lung adenocarcinoma, with immunohistochemical results (CK5/6(−), P40(−), TIF-1( +), Napsin-A( +), P53( +), Ki-67(+ , 3%), EGFR( +)), all resected lymph nodes were negative(Fig. 2). Subsequently, the EGFR gene test was performed, and the result manifested the pathology as a primary T790M (Ex20 T790M: p.T790M (c.2369C > T)) mutation. Aggressive surgical treatment or stereotactic body radiation therapy was considered not feasible for this case, and the patient refused to undergo chemotherapy. After multidisciplinary discussions and communications with the patient, the target therapy was adopted as the follow-up treatment. In March 2019, the patient was treated with 80 mg osimertinib on a daily basis (According to the drug instructions, osimertinib is suitable for patients with advanced non-small cell lung cancer with primary EGFR-T790M mutation). After 3 months of therapy, the chest CT showed that most GGNs in the right lung were partially absorbed. Therefore, the osimertinib treatment was continued. Until the last follow-up in July 2021, the reviewed chest CT exhibited that most GGNs in the right lung were completely absorbed, only one GGN left was radiologically the same as before (Fig. 3). We suggested the patient to excise this single lesion, but she refused. So far, she remains receiving the osimertinib treatment. We will closely follow up checking the residual GGN. If the nodule progresses, it will be strongly recommended to perform thoracoscopic surgical resection to obtain the information about relevant gene mutation. If the patient still refuses surgery, SBRT can be taken.

## Discussions and conclusions

With the improvement of public health awareness and the popularization of thin-section CT, the detective rate of SMPLC with mGGNs has been increasing [16, 17]. But it remains hard to distinguish SMPLC from intrapulmonary metastases without surgical procedure, while a definitive diagnosis is critical because the therapeutic approaches for these two distinct diseases are entirely different [18, 19]. Currently there is no uniform standard of surgical indication for SMPLC with mGGNs [20]. One- or two-stage surgical resection has been proven as the most effective treatment [21, 22]. However, GGNs in both lungs are mostly impossible to treat with complete surgical resection, therefore it is critical to explore the effective adjuvant therapy.

It were reported that various studies on the treatment strategy of multiple ground-glass opacities with pulmonary adenocarcinoma have performed adjuvant therapies, such as Noriko et al. [23] and Yang et al. [5] both tried the chemotherapy regimens (amrubicin,pemetrexed, cisplatin), but obtained no significant effects. Wu et al. [11] reported that GGNs might not be sensitive to anti-PD-1/PD-L1-based monotherapy or combinatorial therapy. Moreover, the SBRT can be considered to achieve local control [9, 10]. According to the American National Comprehensive Cancer Network guidelines for Non-Small Cell Lung Cancer (NSCLC) in 2021 [24], the Osimertinib is recommended to be used as an adjuvant treatment for the EGFR-mutated early-stage NSCLC. However, it remains unclear whether the Osimertinib show effects on residual mGGNs after resection for dominant lesion harboring EGFR-mutation. Recently, Ye et al. [25] reported a successful case of treatment for multiple pulmonary nodules. They performed the surgical resection for the gefitinib-insensitive lesion, and continued the target therapy for sensitive lesions. Cheng et al. [26] reported the impact of postoperative EGFR-TKIs treatment on residual GGN lesions after resection for lung cancer.

In this case, from the preoperative chest CT, the SMPLC was firstly detected, and the indication of left lung lesion for surgery was clear. Postoperative pathology verified the adenocarcinoma for the dominant lesion and the genetic test identified a mutation in T790M gene of the EGFR. The patient refused to undergo partial resection of the right lung lesions. We diagnosed it as SMPLC, but we could not completely rule out the existence of intrapulmonary metastasis. Then, osimertinib was selected as the follow-up treatment, and the therapeutic effect was relatively satisfactory in the end.

Through this case and previous reports, we can find that EGFR-TKI is effective at the treatment of lung cancer with GGNs. Furthermore, we consider that EGFR-TKI treatment for GGNs should meet the following notes: 1) GGNs are in both lungs and cannot be completely resected; 2) Pathological examination confirmed the disease as lung adenocarcinoma; 3) Genetic test revealed the mutation in EGFR gene. Besides, there remain questions to ponder, for example, how long the treatment cycle is? When to finalize the treatment so that the patient will benefit the most? These questions need more researches to set a superior standard of care.

## Data Availability

All data generated or analyzed during this study are included in this article.
